# Perceived benefits of implementing a caregiver-led training programme for caregivers of children with cerebral palsy in rural Mangochi, Malawi: a qualitative exploration

**DOI:** 10.11604/pamj.2026.53.77.50018

**Published:** 2026-02-12

**Authors:** Takondwa Connis Bakuwa, Gillian Saloojee, Wiedaad Slemming

**Affiliations:** 1 Department of Rehabilitation Sciences, Kamuzu University of Health Sciences, Blantyre, Malawi,; 2 Department of Paediatrics and Child Health, Division of Community Paediatrics, Faculty of Health Sciences, University of Witwatersrand, Johannesburg, South Africa,; 3 Department of Physiotherapy, Faculty of Health Sciences, University of Witwatersrand, Johannesburg, South Africa,; 4 Department of Paediatrics and Child Health, Children´s Institute, Faculty of Health Sciences, University of Cape Town, Johannesburg, South Africa

**Keywords:** Caregiver-led training, implementation, perceived benefits, cerebral palsy, community-based

## Abstract

**Introduction:**

caregiver-led training programmes aim to address gaps in rehabilitation services for children with cerebral palsy (CP) in rural Malawi. This qualitative study explored the benefits of implementing such a programme in a rural setting in Malawi.

**Methods:**

a qualitative study was conducted in August 2023, involving in-depth interviews with 11 caregivers, four expert caregivers, and two physiotherapists who participated in the Malamulele Onward Carer-2-Carer Training Programme. Data were analyzed thematically using four constructs of the RE-AIM framework: reach, effectiveness, adoption, and maintenance.

**Results:**

caregivers reported improved knowledge of CP, enhanced caregiving skills, and positive shifts in attitudes, contributing to better well-being for themselves and their children. The programme fostered family engagement, dispelled myths about CP, and increased community awareness. Notably, caregivers trained by fellow caregivers described a stronger sense of empowerment, increased confidence in their caregiving abilities, and greater willingness to share knowledge within their communities. Physiotherapists observed that caregiver-led facilitation encouraged peer support and collective problem-solving. Additionally, physiotherapists gained confidence in managing CP and experienced reduced workloads as caregivers took on more responsibilities. Potential for maintenance of caregiving practices and community engagement was evident; however, long-term sustainability and scalability will require structured policy-level support.

**Conclusion:**

this qualitative study demonstrates caregivers´ and physiotherapists´ perceived improvements in knowledge, skills, attitudes, and well-being, alongside enhanced social inclusion and reduced stigma associated with participation in the caregiver-led training programme. These findings suggest that caregiver-led approaches may hold potential for scalability and sustainability in low-resource settings, offering an accessible strategy for addressing rehabilitation gaps.

## Introduction

Cerebral palsy (CP) is a childhood-onset motor disability that affects movement and posture. The motor impairments often limit activity and participation in daily ongoing therapy crucial for improving independence and quality of life [1]. However, children with CP in Low- and Middle-Income Countries (LMICs) like Malawi face numerous barriers to accessing essential rehabilitation services [2-4]. The scarcity of healthcare professionals, including physiotherapists, along with logistical and financial challenges, results in inadequate and delayed care [4,5]. In rural settings, these constraints are particularly pronounced, leaving many children with CP without regular access to rehabilitation services necessary to optimise function and participation [6,7]. In response to these access constraints, caregiver training programmes have emerged in LMICs as a promising solution to bridge the gap in services, particularly for families in rural areas who are not able to consistently access professional therapy services [8,9]. Caregiver training programmes aim to empower parents and caregivers by providing them with the necessary skills and knowledge to carry out therapeutic activities with their children in daily routines [10,11]. This approach not only reduces the need for frequent clinical visits but also enables caregivers to integrate therapy into the home environment, where children spend the majority of their time [9]. Studies from LMICs have demonstrated the benefits of such programmes, particularly in improving caregiver self-efficacy, reducing stress, and promoting better developmental outcomes in children with disabilities [8,12]. Studies have also shown that caregiver training contributes to notable improvements in child participation in daily activities and increased caregiver confidence in managing their child's needs [6-10]. Despite this growing evidence base, most caregiver training approaches in LMICs remain professional-led, relying heavily on already limited rehabilitation personnel [3]. This dependence raises important questions about the long-term scalability, sustainability, and equity of such models in low-resource, rural contexts where professional availability is severely constrained [3,13].

There remains a critical gap in understanding how caregiver training programmes can be delivered in ways that reduce reliance on professionals while preserving quality, acceptability, and meaningful outcomes for families. Building on this foundation, a further innovation of caregiver-led training is emerging not only to bridge persistent access barriers but also to harness the unique advantages of peer support. In this approach, training is facilitated by expert caregivers, individuals who have acquired lived experience and practical expertise in raising a child with a disability. These peer facilitators share insights, strategies, and encouragement in ways that are often more relatable and contextually grounded than those delivered by professionals. Importantly, caregiver-led training preserves the empowering principles of traditional caregiver training but amplifies them through peer-to-peer engagement, shared experience, and community solidarity [14]. In Malawi, where the healthcare infrastructure is already strained and rehabilitation professionals are few, especially in rural districts like Mangochi, this caregiver-led training approach may offer particular advantages [15]. The country continues to register a high prevalence of childhood disabilities, including CP [5], yet access to specialist care remains limited outside major urban centres. Caregiver-led training offers a sustainable, community-based model of service delivery that would not only expand reach but also foster local capacity, social support, and inclusion [16]. This qualitative study was conducted following a feasibility trial of the Malamulele Onward Carer-to-Carer Training Programme (MOC2CTP) implemented in rural Mangochi, Malawi. The trial compared two delivery approaches: a caregiver-led arm, in which expert caregivers facilitated sessions for fellow caregivers, and a physiotherapist-led arm, in which professionals facilitated sessions for caregivers. Quantitative findings from the trial demonstrated measurable improvements in both caregiver- and child-level outcomes using standard assessment tools [17]. The present qualitative study addresses the identified gap by exploring caregivers´ and physiotherapists´ perceptions of the benefits of implementing a caregiver-led training programme, offering insight into how and why this approach may contribute to improved experiences, empowerment, and service delivery for children with CP and their caregivers in rural, low-resource settings.

## Methods

**Study design:** this was a descriptive phenomenological qualitative study that used in-depth interviews (IDIs) to explore the lived experiences and perceived benefits of a caregiver-led training programme among caregivers of children with CP and physiotherapists involved in its implementation. The approach was considered appropriate for capturing participants´ perspectives while remaining closely grounded in their accounts, in line with the exploratory aims of the study.

**Study setting:** the study was conducted at a community-based organisation (CBO) called Tiyende Pamodzi which is found in rural Mangochi district, southern region of Malawi. This grassroots non-profit organisation provides physiotherapy, assistive devices, and nutritional support for children with disabilities in collaboration with the district hospital and other partners. By January 2023, over 400 children, mainly with cerebral palsy (CP), were served, supported by one physiotherapist overseeing 12 satellite centres. Between January and August 2023, 83 caregivers participated in a feasibility trial of the Malamulele Onward Carer-to-Carer Training Programme (MOC2CTP), a seven-module initiative designed to empower caregivers in CP management. Modules cover CP understanding, positioning, feeding, play, communication, vision, and integration of therapy into daily routines to improve caregiver knowledge and child outcomes. Delivered by trained expert caregivers, who are themselves parents/ caregivers of children with CP, the programme combines psychosocial support, discussions, practical demonstrations, and group activities in 2-2.5 hour workshops (Table 1).

**Table 1:** overview of the seven modules of the adapted Malamulele onward carer-to-carer training programme implemented in Mangochi District, Malawi, from January to August 2023 (N=83)

Module	Content
1	Introduction to CP: what is it and how does it affect my child
2	CP as a way of life: looking after my child throughout the day
3	Getting active: getting my child’s body ready to move throughout the day
4	Eating and drinking: making mealtimes safe and comfortable for my child
5	Communication: my child and I understand each other
6	Cerebral visual impairment: understanding where and what my child can see
7	Play: unlocking my child's potential

CP: cerebral palsy

**Implementation of the training programme in Malawi:** the Malamulele Onward Carer-to-Carer Training Programme (MOC2CTP) was implemented for the first time in Malawi as a feasibility randomised controlled trial conducted over seven weeks. In this trial, 83 caregivers of children with CP received the training in two parallel arms allocated randomly in a 1:1 ratio (caregiver-led vs therapist-led arm). Group allocation was stratified according to the Gross Motor Function Classification System (GMFCS), which categorises children with CP into five levels based on mobility, ranging from independent walking (Level I) to severe motor limitations in self-mobility (Level V) [18]. Both groups participated in weekly workshops for seven consecutive weeks, each cohort consisting of approximately ten caregivers. Sessions in the caregiver-led arm were facilitated by four expert caregivers working in pairs, while those in the therapist-led arm were conducted by two physiotherapists with assistance from a trained translator. Each session integrated psychosocial support, group discussions, and practical demonstrations using a picture-based guide and readily available household materials such as pillows, balls, and feeding utensils. All facilitators received structured training before programme delivery. The four expert caregivers completed 30 hours of preparatory instruction under a master trainer, supplemented by refresher workshops and supervised practice. The two physiotherapists, one affiliated with the Tiyende Pamodzi CBO and the other from the district hospital, underwent equivalent training and additional orientation through workshops and remote mentorship. Details of the feasibility trial design and implementation procedures are described more fully elsewhere [14].

**Study participants:** a total of 17 participants were included in the study, comprising six trainers and eleven caregivers. All trainers were included and consisted of four expert caregivers (caregivers of children with cerebral palsy who had been trained to facilitate the MOC2CTP) and two physiotherapists who were similarly trained to provide facilitation and technical support. Eleven caregivers were purposively selected from among the trainees to capture diverse characteristics and experiences. Selection considered variation in caregiver age, marital status, and relationship to the child, as well as the age, sex, and severity of cerebral palsy among the children under their care.

**Data collection:** qualitative data were collected through in-depth interviews (IDIs) with caregivers and physiotherapists. Data were collected approximately four weeks after completion of the training programme. This follow-up period was intended to allow caregivers sufficient time to apply what they had learned and to reflect on the perceived benefits and outcomes of using the newly acquired skills in their home environments. All in-depth interviews were conducted between 26 and 30 August 2023 within the premises of the Tiyende Pamodzi CBO. The interviews were led by a female social worker with a Bachelor´s degree in social work in community development and more than seven years of experience in social work, counselling, and qualitative research in the region. She was assisted by a trained female social work assistant who was fluent in both local languages. To promote comfort and openness among male caregivers, two male health surveillance assistants (HSAs) conducted interviews with the four male participants. Both HSAs had over five years of experience in community-based health and qualitative data collection. None of the interviewers were involved in programme implementation, a deliberate measure to minimise response bias and encourage honest discussion of participants´ experiences. Participants were recruited face-to-face and enrolled in the study after providing informed consent. Interviews continued until no new information emerged, with data saturation achieved after eleven caregiver interviews. All interviews were audio-recorded with participant consent. Field notes and observational memos were taken by the research assistants to complement the transcripts and support interpretation. Each interview lasted approximately 50 minutes. Interview guides were developed with reference to four out of the five constructs of the RE-AIM framework (Reach, Effectiveness, Adoption, Implementation, and Maintenance), as outlined in Table 2. The implementation construct was not included, as it is examined in a separate qualitative study focused on the facilitators and barriers to programme delivery [19]. The RE-AIM framework is a well-established implementation science model that facilitates comprehensive evaluation of an intervention´s benefits and effects across individual, community, and system levels [20-22].

**Table 2:** application of the RE-AIM framework to guide qualitative interviews evaluating the adapted Malamulele onward carer-to-carer training programme in Mangochi District, Malawi, in August 2023 (n=17)

RE-AIM construct	Description of construct	Example interview questions
Reach	The extent to which the programme successfully engaged caregivers, family members, and influenced broader community perceptions of CP	What motivated you to participate? ii) How easy/difficult was your participation? How easy/difficult was it to get caregivers to participate? iii) How did your family or community react when you joined the programme?
Effectiveness	The impact of the programme on caregiver knowledge, skills, confidence, and child well-being	-What are the most important things you learned from the programme? ii) How has your approach to caring for your child changed since completing the training? iii) Have you noticed any improvements in your child's mobility, feeding, or communication? iv) What aspects of the programme were most useful to you?
Adoption	The extent to which caregivers and therapists applied the practices taught in their daily lives	i) Which skills or techniques from the training do you use regularly at home? -ii) How easy/difficult has it been to apply these new ideas in your daily routine? iii) How confident do you feel in using the techniques you learned? iv) How has your interaction with other caregivers changed since the training programme?
Maintenance	The potential for sustained use of programme skills and long-term benefits for caregivers and children	i)What support do you think would help you continue using what you learned/ implementing the programme? ii) What lasting changes have you seen in your child or family as a result of the training? iii) What could be done to keep this programme going for more caregivers in the future?

**Reflexivity:** the authors are qualified physiotherapists with expertise in CP and disability research. TB (principal investigator) has over five years of experience with CP but was engaging with the MOC2CTP for the first time and had no prior relationships with the caregivers or the CBO representatives. WS and GS have extensive experience in disability research, with GS leading the development of the MOC2CTP and assessing its regional applicability. To ensure reflexivity, the research team maintained reflexive memos throughout data collection and analysis, engaged in regular team discussions to review emerging themes, and conducted iterative analytic reflection to ensure interpretations remained grounded in participants´ narratives. These steps helped the team leverage their expertise while minimising bias and enhancing analytical transparency and trustworthiness.

**Data management and analysis:** audio-recorded data were transcribed verbatim in Yao and Chichewa. The transcripts were then returned to physiotherapists for verification, and selected discussions were revisited with caregivers to confirm the accuracy and interpretation of their accounts. The transcripts were then translated entirely into English and imported into NVivo version 12 (QSR International) for thematic analysis. All digital data, including audio recordings, transcripts, and translated files, were stored on password-protected computers with access restricted to the research team. Hard-copy study materials were securely stored in locked cabinets, and all data were anonymised prior to analysis using unique participant identification codes. A thematic analysis was conducted using the four selected constructs of the RE-AIM framework, Reach, Effectiveness, Adoption, and Maintenance. We used an abductive approach, which combines both inductive and deductive reasoning [23]. In this process, TB started by looking closely at the data without any specific framework in mind (inductive coding). Patterns and themes were identified through a careful reading of the transcripts and analysis of participants´ narratives. To ensure coding consistency and enhance interpretive credibility, one transcript was independently coded by WS and GS. Coding discrepancies were identified through comparison of independently coded transcripts and were discussed in team analytic meetings. Differences in interpretation were resolved through reflexive dialogue, with reference to the original data and the study objectives, until consensus was reached on code definitions and theme boundaries. Patterns and themes were identified through detailed reading and examination of the transcripts, after which they were organised under the RE-AIM constructs (deductive coding), using the framework to guide interpretation. More specific subthemes were then developed within each RE-AIM construct to capture nuances and refine the structure of the analysis. This approach enabled openness to new insights while maintaining a structured interpretation aligned with an established implementation framework.

**Ethical considerations:** ethical approval for this study was obtained from the Human Research Ethics Committee (Medical) of the University of the Witwatersrand, South Africa (M220924), and the College of Medicine Research Ethics Committee, Kamuzu University of Health Sciences, Malawi (P.04/22/3608). The study objectives were clearly explained to all participants, including the researchers´ intent to use their insights to understand perceived benefits and inform future implementation of the caregiver-led training programme. Participation was voluntary, and written informed consent was obtained from all participants. For individuals unable to write, consent was documented using a thumbprint. To ensure confidentiality, no personal identifiers were included in transcripts or reports, and access to study data was limited to the research team.

## Results

Findings from the interviews revealed several perceived benefits of implementing the caregiver-led training programme. The results are organised according to the four RE-AIM constructs that were selected for this study: reach, effectiveness, adoption, and maintenance.

**Reach:** the programme successfully engaged participants and had a broader impact beyond individual caregivers. This broader impact was reflected in reported changes among immediate family members and within caregivers´ households, particularly through increased involvement of spouses, grandparents, and older siblings in child care activities, as well as shifts in attitudes towards disability. Four key themes emerged related to reach: i) the programme met participant expectations; ii) family members became actively involved; iii) disability-related myths were dispelled, and four (4) participants saw a potential for programme expansion. Table 3 highlights the quotes.

**Table 3:** subthemes and illustrative quotations related to the “Reach” construct of the RE-AIM framework from qualitative interviews conducted in Mangochi District, Malawi, in August 2023 (n=17)

Sub-theme	Quote	Perspective
Expectations of participants were met	“*We have learnt that CP is not a disease that can be cured... it all depends on how the family is living. It has nothing to do with witchcraft like we believed.”*	Caregiver 1, caregiver-led arm
Family members became actively involved	“*It has changed my family members a lot... they follow what we are learning and they are more interested each day.”*	Caregiver 2, therapist-led arm
Disability-related myths were dispelled	“*We have learnt that CP is not a disease that can be cured... it all depends on how the family is living. It has nothing to do with witchcraft like we believed.”*	Caregiver 3, caregiver-led arm
Participants saw potential for programme expansion	“*The CP programme should continue... to reach other people, I think we can reach even more families more easily!”*	Expert caregiver 4

This table presents qualitative findings illustrating how participants perceived the “reach” of the Malamulele Onward Training implemented in 2023 in a rural setting in Malawi. They highlight its ability to engage caregivers and influence families and communities. Quotes were drawn from interviews with caregivers and expert caregivers in both the therapist-led and caregiver-led study arms, highlighting shifts in expectations, family involvement, and community awareness about cerebral palsy

**Effectiveness:** the programme led to meaningful changes for participants in four key areas: 1) knowledge of CP; ii) practical caregiving skills; iii) attitudes toward CP, and iv) caregiver and child well-being. Caregivers reported a deeper understanding of CP, more positive attitudes, and reduced stress and self-blame. Participants also reported acquiring practical caregiving skills that made everyday routines such as feeding, positioning, and communication more effective and less stressful. Several caregivers observed that these changes in their own confidence and competence were mirrored by noticeable improvements in their children´s function, mobility, and participation in daily life. Sharing knowledge strengthened CP understanding for both the expert caregivers and physiotherapists. For the physiotherapists, this also improved their skills in lesson planning and tailored support. Moreover, the training promoted peer accountability, strengthened parent-child bonds, and contributed to an overall better quality of life. Evidently, the peer support, opportunities for practice, shared learning experiences and ongoing reinforcement of skills helped participants consolidate knowledge and build confidence in applying new strategies. Table 4 provides illustrative quotes from caregivers, expert caregivers, and physiotherapists.

**Table 4:** subthemes and illustrative quotations related to the “Effectiveness” construct of the RE-AIM framework from qualitative interviews conducted in Mangochi District, Malawi, in August 2023 (n=17)

Sub-theme	Quote	Perspective
Improved knowledge of CP	“*Before we just thought our children maybe were bewitched or cursed or something. But now we know what may have caused their disability. It was never my fault.”*	Caregiver 4, caregiver-led arm
	“*I also have a child with CP... I only got to understand CP better when I was teaching it. A lot of the things make more sense now that I am explaining them to my fellow mothers.”*	Expert caregiver 2
	“*The lesson of CVI (Cerebral Visual Impairment)... we didn't learn in university. This was my first time learning and teaching it”*.	Physiotherapist 1
Improved practical skills	*“When he wakes up... I proceed with what we learnt, massaging and all. I know how to do it in a way that relaxes his body.”*	Caregiver 5, caregiver-led arm
	*“Before the programme I did not really talk to him much but now we communicate and he can laugh with me.”*	Caregiver 4, therapist-led arm
	“*My skills have indeed improved... I am also sharing with friends*.”	Expert Caregiver 1
	“*We learned practical ways... how the planning should be to make the learning relatable*.”	Physiotherapist 2
Positive attitudes towards CP	“*The way I see my child now is different... I don't walk around sad anymore. I have hope*.”	Caregiver 3, caregiver-led arm
	“*There are some things we knew but didn't do...but now we know why they are important, we now do them with intention*.”	Caregiver 6, therapist-led arm
	“*One day my friends will ask, how about you? So that encouraged me to lead by example and do my best with my child too*.”	Expert Caregiver 2
	“*Working with CP was overwhelming... now I feel enlightened and capable*.”	Physiotherapist 2
Improved caregiver well-being	“*Honestly my quality of life improved a lot and my mind is at rest... now I know it was never my fault*.”	Caregiver 7, caregiver-led arm
	“…*but now I manage to walk freely and my life has changed. I am able to go to work and when am back I just relax*.”	Caregiver 10, therapist-led arm
Improved child functioning & Participation	“*My child is now able to do some things by himself; sometimes he wakes up by himself, crawls by himself, we help him sit, he crawls to draw water from the bucket*.”	Caregiver 1, caregiver-led arm
	“*It has helped him a lot because a lot of things now, and even when bathing him he smiles and we communicate with him. Even before feeding him, we massage his cheeks so as to prepare him and all is going well because after doing this he eats quite well*.”	Caregiver 4, therapist-led arm
	“…*now my child is able to show some gestures which his friends can understand when he needs something*.”	Caregiver 11, caregiver-led arm

**Adoption:** three main themes emerged related to the adoption of practices: i) adoption of play-based communication; ii) embracing new caregiving practices, and iii) continued home practice. Most participants across both training arms reported actively deciding to integrate these practices into their daily routines, demonstrating uptake of the training content. Caregivers began using playful interactions to strengthen bonds with their children, several integrated new caregiving techniques into everyday activities, and most established consistent home-based routines. These changes often extended to involve other household members, reflecting participants’ ownership and internalisation of the training content. Table 5 presents illustrative quotes highlighting these changes.

**Table 5:** subthemes and illustrative quotations related to the “Adoption” construct of the RE-AIM framework from qualitative interviews conducted in Mangochi District, Malawi, in August 2023 (n=17)

Sub-theme	Quote	Perspective
Adoption of play-based learning	“*I have learnt to be silly, to pretend to fail to open a bottle top... my child can now laugh with me…*”	Caregiver 5, caregiver-led arm
Embracing new care practices	“*Now I see that... my child seems to be happy. I think the new ways are working well*.”	Expert caregiver 3
Continued home practice	*“After learning each time, I went home and practiced, everyone at home is now getting used to this new way of life.”*	Caregiver 1, therapist-led arm

**Maintenance:** four key themes reflected the potential for continued impact of the programme: i) sustained caregiving practices; ii) continued family involvement and support; iii) increased community awareness and vigilance, and iv) potential for programme sustainability with government support. Although the follow-up period was short (approximately four weeks), participants described factors that signal potential for maintenance, including early establishment of consistent home routines, active involvement of family members, and growing community responsiveness to disability. Physiotherapists also highlighted the importance of government support to integrate and sustain the model through formal structures. Table 6 presents illustrative quotes for each theme.

**Table 6:** subthemes and illustrative quotations related to the “Maintenance” construct of the RE-AIM framework from qualitative interviews conducted in Mangochi District, Malawi, in August 2023 (n=17)

Sub-theme	Quote	Perspective
Sustained caregiving practices	“…*everyone at home is now getting used to this new way of life making the things we learnt as a way of life*.”	Caregiver 9, caregiver-led arm
Family’s continued role in child care	“*Now I manage to walk freely and my life has changed. I can leave my child at home knowing he will be taken care of well*.”	Caregiver 4, therapist-led arm
Sustained community awareness and vigilance	“*We will be more vigilant to rush to the hospital during pregnancy and also when labor starts before nine months*.”	Caregiver 11, caregiver-led arm
Potential for programme sustainability with government support	“*More expert caregivers... should be employed by the government. They can really support service delivery and provide continuous support*.”	Physiotherapist 1

## Discussion

This study adds to the growing body of evidence supporting caregiver-delivered training programmes in low-resource settings, particularly for children with cerebral palsy (CP). While much of the earlier research has focused on programmes delivered by professionals [24-26], findings from this study contribute to the expanding evidence that caregiver-led approaches can have emerging impact beyond individual caregivers to influence families, communities, and even the formal health sector [12,27]. This emerging approach is community-anchored and challenges traditional, provider-led paradigms of rehabilitation. The caregiver-led programme did more than impart knowledge and skills. Caregivers reported a shift from stigma and isolation to shared responsibility and inclusion, demonstrated by their ability to teach others. This aligns with emerging models of empowerment, where those traditionally positioned as recipients of care become agents of change [10]. That caregivers began to act as informal educators within their households and communities suggests perceived diffusion of knowledge that formal services alone often fail to achieve. Importantly, this diffusion was facilitated by the use of local languages and peer facilitation, underscoring the value of cultural and linguistic relevance in intervention design [28-30]. While practical skill acquisition in feeding, positioning, and mobility was a clear outcome, its significance lies in how these practices were embedded in everyday life. Caregivers moved from passive care to proactive rehabilitation. Yet, the value of this transition is not only clinical, reducing the risk of complications such as contractures and pressure sores but also relational. Improved bonding, confidence, and communication transformed caregiving into an affirming experience, with implications for both caregiver and child well-being.

Peer learning emerged as a critical mechanism. Unlike traditional top-down training, the horizontal exchange of experiences fostered solidarity, reduced emotional burden, and created a form of accountability rooted in community relationships [8,31,32]. This peer dynamic, often underestimated, may be a key driver of sustainability. Group-based support not only complements clinical advice but buffers against the chronic stress commonly associated with raising a child with a disability [32]. For physiotherapists, participating in this study prompted reflection on their role in community-based rehabilitation. Although they delivered the same basic training as caregivers in the parallel control arm, they acknowledged that the caregiver-led model could offer a foundation for future task-sharing. Rather than diminishing the physiotherapist´s role, delegating basic training could ultimately allow professionals to concentrate on complex needs, supervision and support [33-35]. This underscores the value of evidence-informed task-sharing, but also the importance of rigorous evaluation to ensure quality, equity, and sustainability in such transitions. A key finding of this study relates to the sustainability of caregiver-led training programmes. Caregivers reported early indications of continued use and sharing of knowledge beyond the formal training, suggesting a shift from programme dependency to community ownership. However, this positive development is accompanied by concerns about long-term feasibility. Reliance on volunteer expert caregivers raises the risk of burnout and exhaustion of the very caregivers who anchor the programme. Physiotherapists also expressed concern about the sustainability of a fully voluntary model, highlighting the need for formal recognition and support for expert caregivers, which could be facilitated through integration into existing community health worker policies and local rehabilitation guidelines. These broader questions about system integration need further study and contextual policy engagement. For caregiver-led models to be effectively scaled, they need to be integrated into existing health systems rather than operate on the margins [36]. This will require adjustments to training curricula, supervision approaches, and financing mechanisms [35,37]. Ensuring the well-being and quality of support provided by expert caregivers should be treated as essential, not just their ability to deliver services at a low cost [33,34].

**Strengths and limitations:** this study provides valuable insights into the perceived benefits of caregiver-led interventions by capturing the experiences of both caregivers and physiotherapists in a rural, resource-limited context. Using the RE-AIM framework strengthened interpretation, while the abductive approach allowed findings to remain grounded in participants´ perspectives. Including both the trainee caregivers and expert caregivers as informants added practical and community-relevant insights. Limitations include the small sample from a single seven-week trial, with a short follow-up period that limits conclusions regarding long-term maintenance and sustainability. Caregiver responses may also have been influenced by social desirability due to ongoing relationships with the community-based organization. Findings should therefore be interpreted as reflecting participants´ perceptions of benefit. Future research should explore similar interventions across diverse contexts and longer follow-up periods to better understand perceived benefits and sustainability.

## Conclusion

This study highlights the wide-ranging benefits of implementing a caregiver-led training programme for families of children with cerebral palsy in rural Malawi. The programme improved caregivers’ knowledge, practical skills, and confidence, while also offering new, context-specific insights for physiotherapists. Caregivers took initiative in applying what they learned, contributed to changing attitudes within families and communities, and supported children’s mobility, interaction, and inclusion. These outcomes extended beyond individual gains to foster caregiver well-being, reduce stigma, and promote broader social participation. In summary, the findings reinforce the value of caregiver-led training not only as an intervention for individual families but as a transformative approach to community-based rehabilitation. Its success depends not just on the content delivered, but on who delivers it, how it aligns with daily realities, and whether systems are willing to embrace more participatory, community-rooted forms of care. With further support and integration into the health system, caregiver-led models could expand access to essential rehabilitation services. Future research should explore how to formalise and sustain the role of expert caregivers without compromising their grassroots strengths, and assess long-term outcomes for children, families, and the broader health system.

### What is known about this topic

- Caregivers of children with cerebral palsy often experience stress, anxiety, and depression, and training can reduce these burdens;
- Skills-based training programmes for caregivers of children with cerebral palsy (CP) improve daily care, feeding, positioning, and mobility outcomes for children;
- Such programmes also reduce caregiver stress and promote confidence, though most evidence comes from professional-led interventions delivered by therapists or rehabilitation specialists.

### What this study adds

- This study provides unique insights into peer-facilitated, caregiver-led training programme delivery in a rural African context, demonstrating how locally trained caregivers can act as both learners and facilitators to support culturally grounded, community-rooted implementation;
- It demonstrates that caregiver-led skills training can achieve benefits comparable to professional-led approaches, leading to improvements in caregiver confidence, competence, and children’s functional participation;
- It also highlights the value of bidirectional learning between caregivers and physiotherapists, where task sharing fostered professional reflection, peer mentorship, and culturally grounded rehabilitation practices, showing how caregiver-led training can catalyse emerging social change, reducing stigma, promoting inclusion, and providing a foundation for community-rooted and potentially sustainable rehabilitation in low-resource settings.
